# Dynamics of chaotic system based on circuit design with Ulam stability through fractal-fractional derivative with power law kernel

**DOI:** 10.1038/s41598-023-32099-1

**Published:** 2023-03-28

**Authors:** Naveed Khan, Zubair Ahmad, Jamal Shah, Saqib Murtaza, M. Daher Albalwi, Hijaz Ahmad, Jamel Baili, Shao-Wen Yao

**Affiliations:** 1 Department of Mathematics, City University of Science and Information Technology, Peshawar, Khyber Pakhtunkhwa 25000 Pakistan; 2 Department of Mathematics and Physics, University of Campania “Luigi Vanvitelli”, 81100 Caserta, Italy; 3 Department of Mathematics, Faculty of Science, King Mongkut’s University of Technology Thonburi (KMUTT), 126 Pracha Uthit Rd., Bang Mod, Thung Khru, Bangkok, 10140 Thailand; 4 Yanbu Industrial College, The Royal Commission for Jubail and Yanbu, Yanbu, 30436 Saudi Arabia; 5 Operational Research Center in Healthcare, Near East University, Near East Boulevard, 99138 Nicosia/Mersin 10, Turkey; 6 Section of Mathematics, International Telematic University Uninettuno, Corso Vittorio Emanuele II, 39, 00186 Rome, Italy; 7 Department of Computer Engineering, College of Computer Science, King Khalid University, Abha, 61413 Saudi Arabia; 8 Higher Institute of Applied Science and Technology of Sousse (ISSATS), University of Souse, Cité Taffala (Ibn Khaldoun), 4003 Sousse, Tunisia; 9 School of Mathematics and Information Science, Henan Polytechnic University, Jiaozuo, 454000 China

**Keywords:** Applied mathematics, Computational science, Electrical and electronic engineering

## Abstract

In this paper, the newly developed Fractal-Fractional derivative with power law kernel is used to analyse the dynamics of chaotic system based on a circuit design. The problem is modelled in terms of classical order nonlinear, coupled ordinary differential equations which is then generalized through Fractal-Fractional derivative with power law kernel. Furthermore, several theoretical analyses such as model equilibria, existence, uniqueness, and Ulam stability of the system have been calculated. The highly non-linear fractal-fractional order system is then analyzed through a numerical technique using the MATLAB software. The graphical solutions are portrayed in two dimensional graphs and three dimensional phase portraits and explained in detail in the discussion section while some concluding remarks have been drawn from the current study. It is worth noting that fractal-fractional differential operators can fastly converge the dynamics of chaotic system to its static equilibrium by adjusting the fractal and fractional parameters.

## Introduction

In recent years, chaos theory has risen its importance, and numerous investigations on chaotic systems have been observed. Different chaotic systems^1,2^ have been studied, particularly hidden^3^ and multi-stability attractors^4^. Chaos-based applications is a hot topic in science and engineering. Oscillators^5^, steganography^6^, synchronization^7^, control^8^, and parameter estimation have all employed chaotic systems. Some chaotic systems have a unique feature. They can have two or more coexisting attractors^9^. Every attractor can be achieved because of the same range of parameters, depending on the initial condition chosen. Multistable chaotic systems^10^ are such systems, and they have gotten a lot of attention in the recent decade because of their potential applications^11^. Several parameters of a multistable dynamical system are particularly sensitive to noise, initial conditions, and system parameters^12^. Although multi-stability makes some engineering fields more challenging, such as bridge vibration and wing design, chaotic systems with multi-stability are extremely beneficial in secure communication^13^. The appearance of hidden attractors has been associated with multi-stability^14^. Stable equilibrium systems and systems with hidden attractors are examples of multistable systems^15^. In multistable systems, self-excited attractors can be found using the typical computational process, but hidden attractors cannot be predicted through the typical computational approach^16^.

In recent decades, fractional calculus has been a significant mathematical technique used to represent critical challenges in various areas, including science, technology, and engineering such as, optimal power flow problems^17^, nonlinear output-error systems^18^, recommender systems with chaotic ratings behavior^19^, parameter estimation of nonlinear control autoregressive systems^20^, power management involving wind-load chaos and uncertainties^21^, Schrodinger equations^22,23^ and shallow water waves^24^. The power-law function is involved in the Liouville–Caputo fractional derivative. As a result, it's common that the physical meaning of the fractional derivative is elusive or non-existent. On the other hand, many researchers identified the physical significance of fractional derivatives^25–33^. The nonlocality property of fractional derivatives is useful in predicting certain materials' memory and heredity properties in certain circumstances. As a result, the fractional derivative^34–37^ can be defined physically as a memory search. Scholars from virtually all fields of science, technology, and engineering have been studying non-local operators of differentiation over the past few decades since they have the capability to incorporate more complex phenomena. Fractional order chaotic systems have also been a highly prominent and attractive topic in recent years. They've been studied by many researchers^38^. Chaotic systems, particularly fractional order-based chaotic systems^39^, have influenced attention as circuit design capability has improved with the introduction of integrated circuits^40^.

The more complex the physical problems, the more complex the mathematical differentiation operators that were developed. In order to perform a single differentiation, the fractal-fractional operator combines fractional differentiation with fractal derivative. These physical processes, for example, exhibit traits of a fractal nature. There is a sort of fractal derivative that may be found in practical mathematics, and it is one in which the variable is scaled in accordance with t^a^. We thought that by coming up with this nonstandard derivative, we would be able to replicate aspects of the physical world that was no longer governed by the conventional physical rules^26^. Two parameters representing power-law, exponential decay, or Mittag–Leffler memory processes were presented by Atangana^41^, and the order parameter order stands for fractal derivative, which can be used to define the fractal dimension. This new set of derivatives also included an order parameter that stands for fractal derivative. Some of the previous work that has been presented in this new field has been applied either to a system of fractional ordinary differential equations or, in a few exceptional cases, to a straightforward equation for heat or diffusion^42–47^.

Motivated from the above literature, it have been found that fractional and fractal-fractional differential operator is a best tool to include the memory effect, crossover behavior and fractal characteristics all at once. Thus, there is a need to modify the existing chaotic systems through fractional or fractal-fractional differential operators of various singular and non-singular kernels. Therefore, in this research we have considered a chaotic system which is based on a circuit design with the following considerations:

- The considered chaotic problem is modelled in terms of nonlinear system of ODEs.
- The integer ODEs system is generalized through the fractal-fractional differential operator of power law kernel.
- The system dissipation and equilibria have been calculated.
- The existence and uniqueness of the solutions have been proved.
- The Ulam stability have been proved.
- Numerical algorithm for the non-linear fractal-fractional system have been stated.
- The obtained graphical results have been portraited through 2D and 3D phase portraits.

## Mathematical preliminaries

This section provides an overview of some essential ideas and prepositions that will be used in subsequent parts of the investigation^41,48,49^.

### **Definition 2.1**

If $g\left( \tau \right)$ is differentiable on $\left( {a,b} \right)$ with fractional order α and fractal order $\beta$ and $g\left( \tau \right)$ is differentiable on the opened interval $\left( {a,b} \right)$, then the Fractal-Fractional derivative of $g\left( \tau \right)$ with fractional order $\alpha$ and fractal order β in Riemann–Liouville sense with power law can be written as:

1 $$_{a}^{FFRL} \wp_{\tau }^{\alpha ,\beta } g\left( \tau \right) = \frac{1}{{\Gamma \left( {m - \alpha } \right)}}\frac{d}{{d\tau^{\beta } }}\int\limits_{a}^{\tau } {g\left( x \right)\left( {\tau - x} \right)^{m - \alpha - 1} dx,}$$

where $$m - 1 < \alpha \le m,\,\,\,0 < m - 1 < \beta \le m.$$

2 $$\frac{dg\left( x \right)}{{dx^{\beta } }} = \mathop {\lim }\limits_{\tau \to x} \,\frac{f\left( \tau \right) - f\left( x \right)}{{\tau^{\beta } - x^{\beta } }}.$$

### **Definition 2.2**

If $g\left( \tau \right)$ is fractal differentiable on $\left( {a,b} \right)$ with fractal order $\beta$ and $g\left( \tau \right)$ is differentiable on the opened interval $\left( {a,b} \right)$, then the Fractal-Fractional derivative of $g\left( \tau \right)$ with fractional order $\alpha$ and fractal order β in Caputo sense with power law is:

3 $$_{a}^{FFP} \wp_{\tau }^{\alpha ,\beta } g\left( \tau \right) = \frac{1}{{\Gamma \left( {m - \alpha } \right)}}\int\limits_{a}^{\tau } {\frac{dg\left( x \right)}{{dx^{\beta } }}\left( {\tau - x} \right)^{m - \alpha - 1} dx,}$$

where $$m - 1 < \alpha \le m,\,\,\,0 < m - 1 < \beta \le m.$$

4 $$\frac{dg\left( x \right)}{{dx^{\beta } }} = \mathop {\lim }\limits_{\tau \to x} \,\frac{f\left( \tau \right) - f\left( x \right)}{{\tau^{\beta } - x^{\beta } }}.$$

### **Definition 2.3**

The fractal-fractional integral of Power law kernel can be expressed as follows:

5 $${}_{0}^{FFP} I_{\tau }^{\alpha } f(\tau ) = \frac{\beta }{N(\alpha )}\int\limits_{0}^{\tau } {x^{\alpha - 1} } u\left( x \right)\left( {\tau - x} \right)^{\alpha - 1} dx.$$

### **Definition 2.4**

Numerical solution to the Caputo ODE of fractional order is defined as follows^50^:

Consider nonlinear fractional ODE:

6 $${}_{0}^{C} \wp_{t}^{\alpha } z(\tau ) = f\left( {\tau ,z(\tau )} \right)\;\;{\text{with}}\;{\text{z}}(0) = z_{0} ,$$

The numerical algorithm for Eq. (6) is as follows:

7 $$z_{n + 1} = z_{0} + \frac{1}{\Gamma (\alpha )}\sum\limits_{j = 0}^{n} {\left[ \begin{gathered} \frac{{h^{\alpha } f\left( {\tau_{j} ,z\left( {\tau_{j} } \right)} \right)}}{{\alpha \left( {\alpha + 1} \right)}}\left\{ {\left( {n + 1 - j} \right)^{\alpha } \left( {n + 2 - r + \alpha } \right) - \left( {n - j} \right)^{\alpha } \left( {n + 2 - r + 2\alpha } \right)} \right\} \hfill \\ - \frac{{h^{\alpha } f\left( {\tau_{j - 1} ,z\left( {\tau_{j - 1} } \right)} \right)}}{{\alpha \left( {\alpha + 1} \right)}}\left\{ {\left( {n + 1 - j} \right)^{\alpha + 1} - \left( {n - j} \right)^{\alpha } \left( {n + 2 - r + \alpha } \right)} \right\} \hfill \\ \end{gathered} \right]} .$$

## Mathematical formulation

In this study, we investigate a new type of chaotic system that was presented in^51^:

8 $$\left. \begin{aligned} & \frac{{dx_{1} }}{d\tau } = z_{1} , \hfill \\ & \frac{{dy_{1} }}{d\tau } = - x_{1} - z_{1} , \hfill \\ & \frac{{dz_{1} }}{d\tau } = ax_{1} + by_{1} - z_{1} + x_{1} y_{1} - cx_{1} z_{1} + 1, \hfill \\ \end{aligned} \right\}.$$

with initial conditions:

9 $$x_{1} \left( 0 \right) = x_{1}^{*} ,\,\,y_{1} \left( 0 \right) = y_{1}^{*} \;\;{\text{and}}\;\;z_{1} \left( 0 \right) = z_{1}^{*} .$$

Here $a,b$ and $c$ are the three non-negative parameters.

The integer order system given in Eq. (8) can be transformed to the fractal-fractional order chaotic system by applying the definition given in Eq. (3). Thus, by applying the procedure given in^41^, Eq. (8) will take the following form:

10 $$\left. \begin{aligned} & {}_{0}^{FFP} \wp_{\tau }^{\alpha ,\beta } = z_{1} , \hfill \\ & {}_{0}^{FFP} \wp_{\tau }^{\alpha ,\beta } = - x_{1} - z_{1} , \hfill \\ & {}_{0}^{FFP} \wp_{\tau }^{\alpha ,\beta } = ax_{1} + by_{1} - z_{1} + x_{1} y_{1} - cx_{1} z_{1} + 1, \hfill \\ \end{aligned} \right\}.$$

The fractional order and fractal dimension are represented by $\alpha$ and $\beta$, respectively, with $0 < \alpha ,\beta \le 1$.

## Dissipation and equilibria

The model in the system (8) is dissipative because:

11 $$\nabla V = \frac{\partial }{{\partial x_{1} }}\left( {\frac{{dx_{1} }}{d\tau }} \right) + \frac{\partial }{{\partial y_{1} }}\left( {\frac{{dy_{1} }}{d\tau }} \right) + \frac{\partial }{{\partial z_{1} }}\left( {\frac{{dz_{1} }}{d\tau }} \right) = - \left( {1 + cx_{1} } \right) < 0.$$

For the fixed points of the chaotic system (8), we consider $\frac{{dx_{1} }}{d\tau } = \frac{{dy_{1} }}{d\tau } = \frac{{dz_{1} }}{d\tau } = 0$. Hence system (8) can be written as:

12 $$\left. \begin{aligned} & 0 = z_{1} , \hfill \\ & 0 = - x_{1} - z_{1} , \hfill \\ &0 = ax_{1} + by_{1} - z_{1} + x_{1} y_{1} - cx_{1} z_{1} + 1, \hfill \\ \end{aligned} \right\}.$$

Here we have only one equilibrium point, i.e., $\left( {x_{1} ,y_{1} ,z_{1} } \right) = \left( {0, - \frac{1}{b},0} \right)$.

## Existence and uniqueness of the fractal-fractional model

The presence of a unique solution to the fractal-fractional model, provided in Eq. (10), is established in this portion of the study. We could reformulate the Eq. (10) in the following manner because the integral is differentiable:

13 $$\left. \begin{gathered} {}_{0}^{RL} \wp_{\tau }^{\beta } \left( {x_{1} \left( \tau \right)} \right) = \beta \tau^{\beta - 1} \left( {\Psi_{1} \left( {\tau ,x_{1} } \right)} \right), \hfill \\ {}_{0}^{RL} \wp_{\tau }^{\beta } \left( {y_{1} \left( \tau \right)} \right) = \beta \tau^{\beta - 1} \left( {\Psi_{1} \left( {\tau ,y_{1} } \right)} \right), \hfill \\ {}_{0}^{RL} \wp_{\tau }^{\beta } \left( {z_{1} \left( \tau \right)} \right) = \beta \tau^{\beta - 1} \left( {\Psi_{1} \left( {\tau ,z_{1} } \right)} \right). \hfill \\ \end{gathered} \right\}.$$

where $$0 < \alpha ,\beta \le 1$$ and,

14 $$\left. \begin{aligned} & \Psi_{1} \left( {\tau ,x_{1} } \right) = z_{1} , \hfill \\ & \Psi_{1} \left( {\tau ,y_{1} } \right) = - x_{1} - z_{1} , \hfill \\ & \Psi_{1} \left( {\tau ,z_{1} } \right) = ax_{1} + by_{1} - z_{1} + x_{1} y_{1} - cx_{1} z_{1} + 1, \hfill \\ \end{aligned} \right\}.$$

We can rewrite system (13) as:

15 $$\left. \begin{aligned} & {}_{0}^{RL} \wp_{\tau }^{\omega } {\rm H}\left( \tau \right) = \beta \tau^{\beta - 1} \Phi \left( {\tau ,{\rm H}\left( \tau \right)} \right), \hfill \\ & {\rm H}\left( 0 \right) = {\rm H}^{*} \hfill \\ \end{aligned} \right\}.$$

where

16 $${\rm H}\left( \tau \right) = \left\{ \begin{gathered} x_{1} \left( \tau \right) \hfill \\ y_{1} \left( \tau \right) \hfill \\ z_{1} \left( \tau \right) \hfill \\ \end{gathered} \right.,\,\,\,\,{\rm H}\left( 0 \right) = \left\{ \begin{gathered} x_{1} \left( 0 \right) \hfill \\ y_{1} \left( 0 \right) \hfill \\ z_{1} \left( 0 \right) \hfill \\ \end{gathered} \right.,\,\,\,\,\,\Phi \left( {\tau ,{\rm H}\left( 0 \right)} \right) = \left\{ \begin{gathered} \Psi_{1} \left( {\tau ,x_{1} } \right) \hfill \\ \Psi_{1} \left( {\tau ,y_{1} } \right) \hfill \\ \Psi_{1} \left( {\tau ,z_{1} } \right) \hfill \\ \end{gathered} \right..\,\,$$

Now, by replacing ${}_{0}^{RL} \wp_{\tau }^{\alpha }$ with ${}_{0}^{C} \wp_{\tau }^{\alpha }$ and applying the fractional integral, we get:

17 $${\rm H}\left( \tau \right) = {\rm H}\left( 0 \right) + \frac{\beta }{\Gamma \left( \alpha \right)}\int\limits_{a}^{\tau } {\kappa^{\beta - 1} \left( {\tau - \kappa } \right)^{\alpha - 1} \Phi \left( {\kappa ,{\rm H}\left( \kappa \right)} \right)} d\kappa .$$

Let ${\rm P}\left( \psi \right)$ be a Banach space of the real-valued continuous functions with supremum norm defined on the interval $\psi = \left[ {0,\Upsilon } \right]$ and $\chi = {\rm P}\left( \psi \right) \times {\rm P}\left( \psi \right) \times {\rm P}\left( \psi \right)$ with the norm $\left\| \Gamma \right\| = \sup \left\{ {\left| {\Gamma \left( \tau \right)} \right|:\,\tau \in \psi } \right\}$.

Now, transform the problem (10) into a fixed-point problem. Define an operator $\coprod :\chi \to \chi$ as:

18 $$\coprod \left( {{\rm H}\left( \tau \right)} \right) = {\rm H}\left( 0 \right) + \frac{\beta }{\Gamma \left( \alpha \right)}\int\limits_{a}^{\tau } {\kappa^{\beta - 1} \left( {\tau - \kappa } \right)^{\alpha - 1} \Phi \left( {\kappa ,{\rm H}\left( \kappa \right)} \right)} d\kappa .$$

For the existence theory, we use the following theorem^52,53^.

### **Theorem 5.1**

*Assume that the operator* $\coprod :\chi \to \chi$ *is completely continuous and the set defined by:*

$\mho \left( \coprod \right) = \left\{ {\Phi \in \chi :\,{\rm H} = \delta \coprod \left( {\rm H} \right);\,\delta \in \left[ {0,1} \right]} \right\}$ *be bounded. Then* $\coprod$ *has a fixed point* $\chi$.

### **Theorem 5.2**

*Let* $\Phi :\psi \times \chi \to {\mathbb{R}}$ *is a continuous function. Then the operator* $\coprod$ *is compact*.

### ***Proof***

Let ${\rm M}$ us a bounded set in $\chi$. Then there is $\vartheta_{\Phi } > 0$ with $\left| {\Phi \left( {\tau ,{\rm H}\left( \tau \right)} \right)} \right| \le \vartheta_{\Phi } ,\,\,\forall {\rm H} \in {\rm M}.$ So for any ${\rm H} \in {\rm M}$ one can get:

19 $$\begin{aligned} \left\| {\coprod \left( {\text{H}} \right)} \right\| & \le \frac{{\beta \vartheta_{\Phi } }}{\Gamma \left( \alpha \right)}\mathop {\max }\limits_{\tau \to \psi } \int\limits_{0}^{\tau } {\kappa^{\beta - 1} \left( {\tau - \kappa } \right)^{\alpha - 1} d\kappa ,} \hfill \\ & \le \frac{{\beta \vartheta_{\Phi } }}{\Gamma \left( \alpha \right)}\mathop {\max }\limits_{\tau \to \psi } \int\limits_{0}^{1} {\varphi^{\beta - 1} \left( {1 - \varphi } \right)^{\alpha - 1} \tau^{\beta + \alpha - 1} d\varphi ,} \hfill \\ & \le \frac{{\beta \vartheta_{\Phi } {\rm T}^{\beta + \alpha - 1} }}{\Gamma \left( \alpha \right)}B\left( {\alpha ,\beta } \right), \hfill \\ \end{aligned}$$

where $B\left( {\alpha ,\beta } \right)$ is the beta function. Thus, ${\rm M}\left( \coprod \right)$ it is uniformly bounded.

Next, for the equicontinuity of the operator $\coprod$, for any $\tau_{1} ,\tau_{2} \in \psi$ and ${\rm H} \in {\rm M},$ we have:

20 $$\begin{aligned} \left\| {\coprod \left( {{\text{H}}\left( {\tau_{1} } \right)} \right) - \coprod \left( {{\text{H}}\left( {\tau_{2} } \right)} \right)} \right\| & \le \frac{{\beta \vartheta_{\Phi } }}{\Gamma \left( \alpha \right)}\mathop {\max }\limits_{\tau \to \psi } \left| {\int\limits_{0}^{{\tau_{1} }} {\kappa^{\beta - 1} \left( {\tau - \kappa } \right)^{\alpha - 1} d\kappa - \int\limits_{0}^{{\tau_{2} }} {\kappa^{\beta - 1} \left( {\tau - \kappa } \right)^{\alpha - 1} d\kappa } } } \right|, \hfill \\ & \le \frac{{\beta \vartheta_{\Phi } }}{\Gamma \left( \alpha \right)}\mathop {\max }\limits_{\tau \to \psi } \left( {\tau_{1}^{\alpha + \beta - 1} - \tau_{2}^{\alpha + \beta - 1} } \right) \to 0\,\,{\text{as}}\,\,\tau_{1} \to \tau_{2} . \hfill \\ \end{aligned}$$

Therefore, $\coprod$ it is equicontinuous. Since $\coprod$ it is a bounded and continuous operator. So, by Arzela-Ascoli theorem is completely continuous.

### **Theorem 5.3**

*Let for all* $\tau \in \psi$ *and* ${\rm H} \in {\mathbb{R}}$, *there is a real number* $\vartheta_{\Phi } > 0$ *with* $\left| {\Phi \left( {\tau ,\coprod \left( \tau \right)} \right)} \right| \le \vartheta_{\Phi }$ . *Then the considered model* (10) *has at least one solution in the given space* $\chi .$

### *Proof*

Consider a set $\mho \left( \coprod \right) = \left\{ {\Phi \in \chi :\,{\rm H} = \delta \coprod \left( {\rm H} \right);\,\delta \in \left[ {0,1} \right]} \right\}$ and show that $\mho$ it is bounded. Suppose ${\rm H} \in \mho$, then ${\rm H} = \delta \coprod \left( {\rm H} \right)$. For $\tau \in \psi$, one can easily obtain:

21 $$\left\| {\rm H} \right\| \le \frac{{\beta \vartheta_{\Phi } {\rm T}^{\alpha + \beta - 1} }}{\Gamma \left( \alpha \right)}B\left( {\alpha ,\beta } \right).$$

Hence $\mho$ is bounded. So, Theorems 5.1 and 5.2$\coprod$ has at least one fixed point. Thus, the considered system (10) has at least one solution.

For further analysis, suppose the following hypothesis:

### **Hypothesis 5.1**

There is a constant $\hbar_{\Phi } > 0$ such that for every ${\rm H},\overline{\rm H} \in \chi$, we have:

22 $$\left| {\Phi \left( {\tau ,{\rm H}} \right) - \Phi \left( {\tau ,\overline{\rm H}} \right)} \right| \le \vartheta_{\Phi } \left| {{\rm H} - \overline{\rm H}} \right|.$$

For uniqueness, we use Banach's Contraction Theorem^52,53^:

### **Theorem 5.4**

*Under Hypothesis* 5.1*and if* $\Xi < 1$. *Then the solution of the considered system* (10) *is unique, where*

23 $$\Xi = \frac{{\beta \hbar_{\Phi } {\rm T}^{\alpha + \beta - 1} }}{\Gamma \left( \alpha \right)}B\left( {\alpha ,\beta } \right).$$

### *Proof*

Let we define $\mathop {\max }\limits_{\tau \to \psi } \left| {\Phi \left( {\tau ,0} \right)} \right| = \omega_{\Phi } < \infty$ such that $\varpi \ge \frac{{\beta {\rm T}^{\alpha + \beta - 1} B\left( {\alpha ,\beta } \right)\omega_{\Phi } }}{{\Gamma \left( \alpha \right) - \beta {\rm T}^{\alpha + \beta - 1} B\left( {\alpha ,\beta } \right)\hbar_{\Phi } }}$.

We show that $\coprod \left( {\Re_{\omega } } \right) \subset \Re_{\omega }$ where $\Re_{\omega } = \left\{ {{\rm H} \in \chi :\left\| {\rm H} \right\| \le \omega } \right\}$. For ${\rm H} \in \Re_{\omega }$, we have:

24 $$\begin{aligned} \left\| {\coprod \left( {\rm H} \right)} \right\| & \le \frac{\beta }{\Gamma \left( \alpha \right)}\mathop {\max }\limits_{\tau \in \psi } \int\limits_{0}^{\tau } {\kappa^{\beta - 1} \left( {\tau - \kappa } \right)^{\alpha - 1} \left( {\left| {\Phi \left( {\tau ,{\rm H}\left( \tau \right)} \right) - \Phi \left( {\tau ,0} \right)} \right| + \left| {\Phi \left( {\tau ,0} \right)} \right|} \right)d\kappa ,} \hfill \\ & \le \frac{{\beta {\rm T}^{\alpha + \beta - 1} B\left( {\alpha ,\beta } \right)\left( {\hbar_{\Phi } \left\| {\rm H} \right\| + \omega_{\Phi } } \right)}}{\Gamma \left( \alpha \right)} \hfill \\ & \le \frac{{\beta {\rm T}^{\alpha + \beta - 1} B\left( {\alpha ,\beta } \right)\left( {\hbar_{\Phi } \varpi + \omega_{\Phi } } \right)}}{\Gamma \left( \alpha \right)} \hfill \\ & \le \Xi . \hfill \\ \end{aligned}$$

Let the operator $\coprod :\chi \to \chi$ define it by (18). Then in view of Hypothesis 5.1, for every $\tau \in \psi$ and for every ${\rm H},\overline{\rm H} \in \chi$, we have:

25 $$\begin{aligned} \left\| {\coprod \left( {\rm H} \right) - \coprod \left( {\overline{\rm H}} \right)} \right\| & \le \frac{\beta }{\Gamma \left( \alpha \right)}\mathop {\max }\limits_{\tau \in \psi } \left| \begin{gathered} \int\limits_{0}^{\tau } {\kappa^{\beta - 1} \left( {\tau - \kappa } \right)^{\alpha - 1} \Phi \left( {\kappa ,{\rm H}\left( \kappa \right)} \right)d\kappa } \hfill \\ - \int\limits_{0}^{\tau } {\kappa^{\beta - 1} \left( {\tau - \kappa } \right)^{\alpha - 1} \Phi \left( {\kappa ,\overline{\rm H}\left( \kappa \right)} \right)d\kappa } \hfill \\ \end{gathered} \right| \hfill \\ & \le \omega \left\| {{\rm H} - \overline{\rm H}} \right\|. \hfill \\ \end{aligned}$$

Hence $\coprod$ is, a contraction from (25). Thus, the integral Eq. (17) has a unique solution, and so does the system (10) has a unique solution.

## Ulam stability

In this section, we develop and present some results on the stability of the model (10). We will consider a small perturbation $\Upsilon \in P\left( \psi \right)$ that depends only on the solution and $\Upsilon \left( 0 \right) = 0$. Further:

26 $$\left| {\Upsilon \left( \tau \right)} \right| \le \varepsilon ,\,\,\,{\text{for}}\,\,\varepsilon > 0,$$

27 $${}^{FFP}\wp_{\tau }^{\alpha ,\beta } \left( {{\rm H}\left( \tau \right)} \right) = \Phi \left( {\tau ,{\rm H}\left( \tau \right)} \right) + \Upsilon \left( \tau \right).$$

### **Lemma 6.1**

*The modified problem's solution is as follows:*

28 $$\left\{ \begin{aligned} & {}^{FFP}\wp_{\tau }^{\alpha ,\beta } \left( {{\rm H}\left( \tau \right)} \right) = \Phi \left( {\tau ,{\rm H}\left( \tau \right)} \right) + \Upsilon \left( \tau \right), \hfill \\ & {\rm H}\left( 0 \right) = {\rm H}^{*} , \hfill \\ \end{aligned} \right.$$

*Satisfies the following relation:*

29 $$\left| {{\rm H}\left( \tau \right) - \left( {{\rm H}\left( 0 \right) + \frac{\beta }{\Gamma \left( \alpha \right)}\int\limits_{a}^{\tau } {\kappa^{\beta - 1} \left( {\tau - \kappa } \right)^{\alpha - 1} \Phi \left( {\kappa ,{\rm H}\left( \kappa \right)} \right)} d\kappa } \right)} \right| \le \left( {\frac{{\beta {\rm T}^{\alpha + \beta - 1} }}{\Gamma \left( \alpha \right)}B\left( {\alpha ,\beta } \right)} \right)\varepsilon = {\mathbb{C}}_{\alpha ,\beta } \varepsilon .$$

### **Theorem 6.1**

*Under* Hypothesis 5.1 and Lemma 6.1, *the integral* Eq. (17) *solution is Ulam-Hyers stable. Consequently, the considered system is Ulam-Hyers stable if* $\Xi < 1$, *where* $\Xi$ *is given by* (23).

### *Proof*

Suppose ${\rm K} \in \chi$ be a unique solution and ${\rm H} \in \chi$ be any solution of (10), then:

30 $$\begin{aligned} \left| {{\rm H}\left( \tau \right) - {\rm K}\left( \tau \right)} \right| & = \left| {{\rm H}\left( \tau \right) - \left( {{\rm K}\left( 0 \right) + \frac{\beta }{\Gamma \left( \alpha \right)}\int\limits_{a}^{\tau } {\kappa^{\beta - 1} \left( {\tau - \kappa } \right)^{\alpha - 1} \Phi \left( {\kappa ,{\rm K}\left( \kappa \right)} \right)} d\kappa } \right)} \right| \hfill \\ & \le \left| {{\rm H}\left( \tau \right) - \left( {{\rm H}\left( 0 \right) + \frac{\beta }{\Gamma \left( \alpha \right)}\int\limits_{a}^{\tau } {\kappa^{\beta - 1} \left( {\tau - \kappa } \right)^{\alpha - 1} \Phi \left( {\kappa ,{\rm H}\left( \kappa \right)} \right)} d\kappa } \right)} \right| \hfill \\ & \quad + \left| \begin{gathered} {\rm H}\left( {{\rm H}\left( 0 \right) + \frac{\beta }{\Gamma \left( \alpha \right)}\int\limits_{a}^{\tau } {\kappa^{\beta - 1} \left( {\tau - \kappa } \right)^{\alpha - 1} \Phi \left( {\kappa ,{\rm H}\left( \kappa \right)} \right)} d\kappa } \right) \hfill \\ - \left( {{\rm K}\left( 0 \right) + \frac{\beta }{\Gamma \left( \alpha \right)}\int\limits_{a}^{\tau } {\kappa^{\beta - 1} \left( {\tau - \kappa } \right)^{\alpha - 1} \Phi \left( {\kappa ,{\rm K}\left( \kappa \right)} \right)} d\kappa } \right) \hfill \\ \end{gathered} \right| \hfill \\ & \le {\mathbb{C}}_{\alpha ,\beta } \varepsilon + \frac{{\beta \hbar_{\Phi } {\rm T}^{\alpha + \beta - 1} }}{\Gamma \left( \alpha \right)}B\left( {\alpha ,\beta } \right)\left\| {{\rm H} - {\rm K}} \right\|. \hfill \\ \end{aligned}$$

We can write the above inequality as:

31 $$\left| {{\rm H}\left( \tau \right) - {\rm K}\left( \tau \right)} \right| \le {\mathbb{C}}_{\alpha ,\beta } \varepsilon + \Xi \left\| {{\rm H} - {\rm K}} \right\|.$$

From (31), we can write:

32 $$\left| {{\rm H}\left( \tau \right) - {\rm K}\left( \tau \right)} \right| \le \left( {\frac{{{\mathbb{C}}_{\alpha ,\beta } }}{1 - \Xi }} \right)\varepsilon .$$

Therefore, the result (32) concludes that the solution of (10) is Ulam-Hyers stable, and therefore this supposed problem is Ulam-Hyers stable as well.

## Numerical scheme for the fractal-fractional model

Consider the system (10)

33 $$\left. \begin{gathered} {}_{0}^{RL} \wp_{\tau }^{\alpha } \left( {x_{1} \left( \tau \right)} \right) = \beta \tau^{\beta - 1} \left( {\Phi_{1} \left( {\tau ,y_{1} } \right)} \right), \hfill \\ {}_{0}^{RL} \wp_{\tau }^{\alpha } \left( {y_{1} \left( \tau \right)} \right) = \beta \tau^{\beta - 1} \left( {\Phi_{2} \left( {\tau ,y_{2} } \right)} \right), \hfill \\ {}_{0}^{RL} \wp_{\tau }^{\alpha } \left( {z_{1} \left( \tau \right)} \right) = \beta \tau^{\beta - 1} \left( {\Phi_{3} \left( {\tau ,y_{3} } \right)} \right). \hfill \\ \end{gathered} \right\}$$

We can also write:

34 $$\left. \begin{gathered} {}_{0}^{C} \wp_{t}^{\alpha } \left( {x_{1} \left( \tau \right)} \right) = \Theta_{1} \left( {\tau ,x_{1} } \right), \hfill \\ {}_{0}^{C} \wp_{t}^{\alpha } \left( {y_{1} \left( \tau \right)} \right) = \Theta_{2} \left( {\tau ,y_{1} } \right), \hfill \\ {}_{0}^{C} \wp_{t}^{\alpha } \left( {z_{1} \left( \tau \right)} \right) = \Theta_{3} \left( {\tau ,z_{1} } \right). \hfill \\ \end{gathered} \right\},$$

Now by replacing ${}_{0}^{RL} \wp_{\tau }^{\alpha }$ with ${}_{0}^{C} \wp_{\tau }^{\alpha }$ and applying the procedure given in Eq. (7), we obtain the numerical algorithm in the following form:

35 $$x_{1} \left( {\tau_{n + 1} } \right) = x_{1} \left( {\tau_{0} } \right) + \frac{1}{\Gamma (\alpha )}\sum\limits_{j = 0}^{n} {\left[ \begin{gathered} \frac{{h^{\alpha } \Theta_{1} \left( {\tau_{j} ,x_{1} \left( {\tau_{j} } \right)} \right)}}{{\alpha \left( {\alpha + 1} \right)}}\left\{ {\left( {n + 1 - j} \right)^{\alpha } \left( {n + 2 - r + \alpha } \right) - \left( {n - j} \right)^{\alpha } \left( {n + 2 - r + 2\alpha } \right)} \right\} \hfill \\ - \frac{{h^{\alpha } \Theta_{1} \left( {\tau_{j - 1} ,x_{1} \left( {\tau_{j - 1} } \right)} \right)}}{{\alpha \left( {\alpha + 1} \right)}}\left\{ {\left( {n + 1 - j} \right)^{\alpha + 1} - \left( {n - j} \right)^{\alpha } \left( {n + 2 - r + \alpha } \right)} \right\} \hfill \\ \end{gathered} \right]} ,$$

36 $$y_{1} \left( {\tau_{n + 1} } \right) = y_{1} \left( {\tau_{0} } \right) + \frac{1}{\Gamma (\alpha )}\sum\limits_{j = 0}^{n} {\left[ \begin{gathered} \frac{{h^{\alpha } \Theta_{2} \left( {\tau_{j} ,y_{1} \left( {\tau_{j} } \right)} \right)}}{{\alpha \left( {\alpha + 1} \right)}}\left\{ {\left( {n + 1 - j} \right)^{\alpha } \left( {n + 2 - r + \alpha } \right) - \left( {n - j} \right)^{\alpha } \left( {n + 2 - r + 2\alpha } \right)} \right\} \hfill \\ - \frac{{h^{\alpha } \Theta_{2} \left( {\tau_{j - 1} ,y_{1} \left( {\tau_{j - 1} } \right)} \right)}}{{\alpha \left( {\alpha + 1} \right)}}\left\{ {\left( {n + 1 - j} \right)^{\alpha + 1} - \left( {n - j} \right)^{\alpha } \left( {n + 2 - r + \alpha } \right)} \right\} \hfill \\ \end{gathered} \right]} ,$$

37 $$z_{1} \left( {\tau_{n + 1} } \right) = z_{1} \left( {\tau_{0} } \right) + \frac{1}{\Gamma (\alpha )}\sum\limits_{j = 0}^{n} {\left[ \begin{gathered} \frac{{h^{\alpha } \Theta_{3} \left( {\tau_{j} ,z_{1} \left( {\tau_{j} } \right)} \right)}}{{\alpha \left( {\alpha + 1} \right)}}\left\{ {\left( {n + 1 - j} \right)^{\alpha } \left( {n + 2 - r + \alpha } \right) - \left( {n - j} \right)^{\alpha } \left( {n + 2 - r + 2\alpha } \right)} \right\} \hfill \\ - \frac{{h^{\alpha } \Theta_{3} \left( {\tau_{j - 1} ,z_{1} \left( {\tau_{j - 1} } \right)} \right)}}{{\alpha \left( {\alpha + 1} \right)}}\left\{ {\left( {n + 1 - j} \right)^{\alpha + 1} - \left( {n - j} \right)^{\alpha } \left( {n + 2 - r + \alpha } \right)} \right\} \hfill \\ \end{gathered} \right]} .$$

## Discussion and results

This section of the article contains graphical representations of the results obtained from the recent study. This study offers the dynamics of a newly designed circuit under the consideration of fractal-fractional derivative of power law kernel. The problem has been analized for both fractional order parameter $\alpha$ and fractal dimension $\beta$. Simulations are performed and results are computed using MATLAB software. For the initial values, we have considered $x_{1} \left( 0 \right) = 5.4,\,\,y_{1} \left( 0 \right) = - 1.8$ and $z_{1} \left( 0 \right) = 3.3$ while $a = 0.1,\,b = 5\,$ and $c = 0.3$.

**Figure 1:**
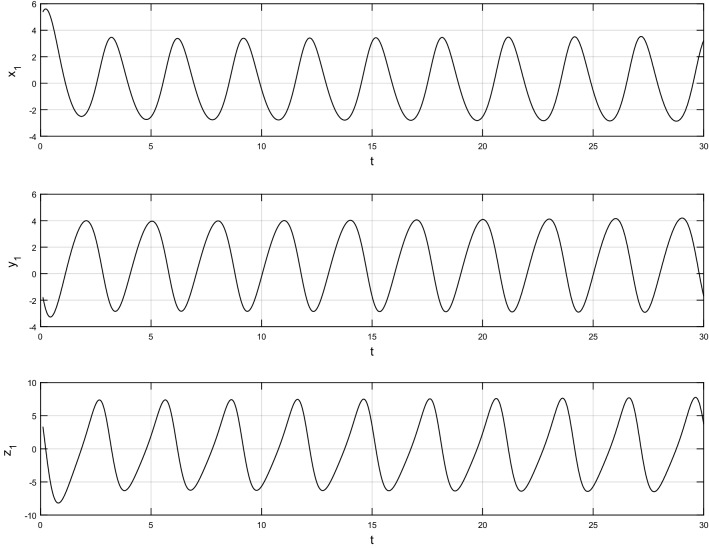
The classical case's chaotic attractor dynamics i.e., *α* = *β* = 1.

**Figure 2:**
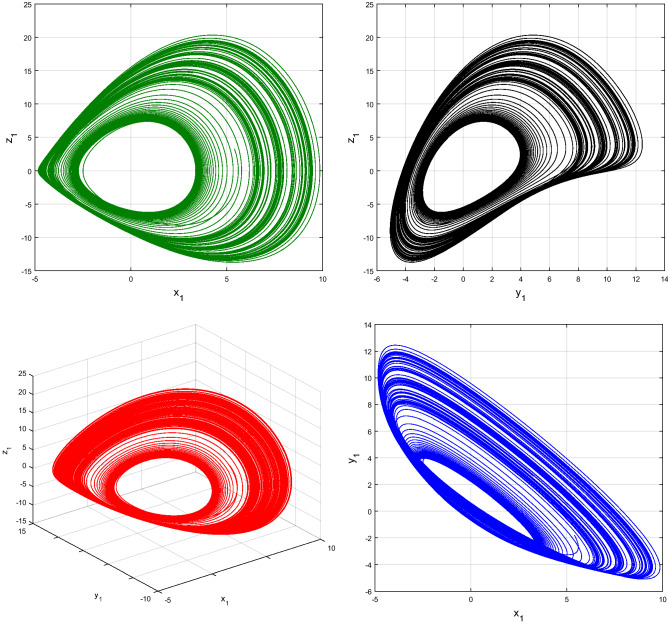
Classical systems' 2D and 3D phase portraits of the chaotic attractor dynamics case i.e., *α* = *β* = 1.

**Figure 3:**
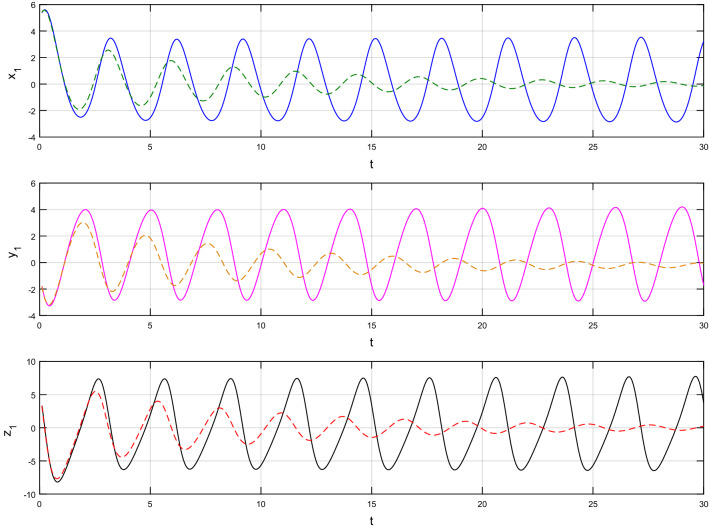
An analysis of the chaotic attractors for this case when *α* = 1 (line) and *α* = 0.95 (dash).

**Figure 4:**
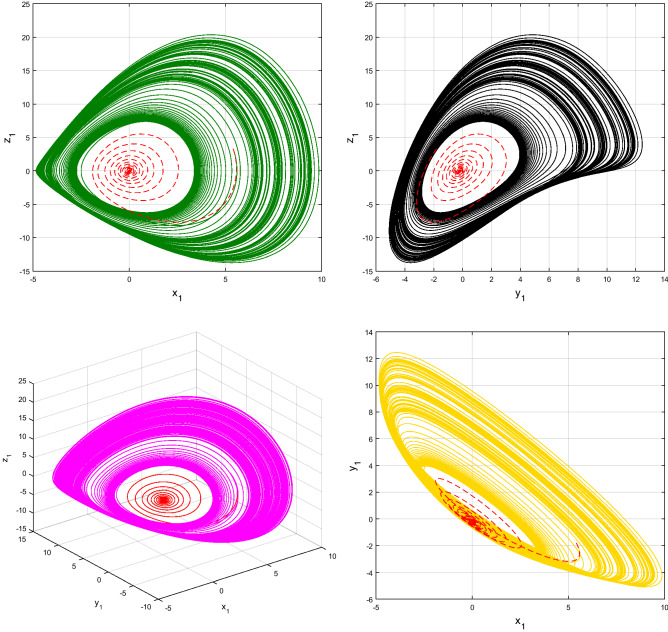
The case's chaotic attractor dynamics, shown in 2D and 3D phase portraits when *α* = 1 (line) and *α* = 0.95 (dash).

**Figure 5:**
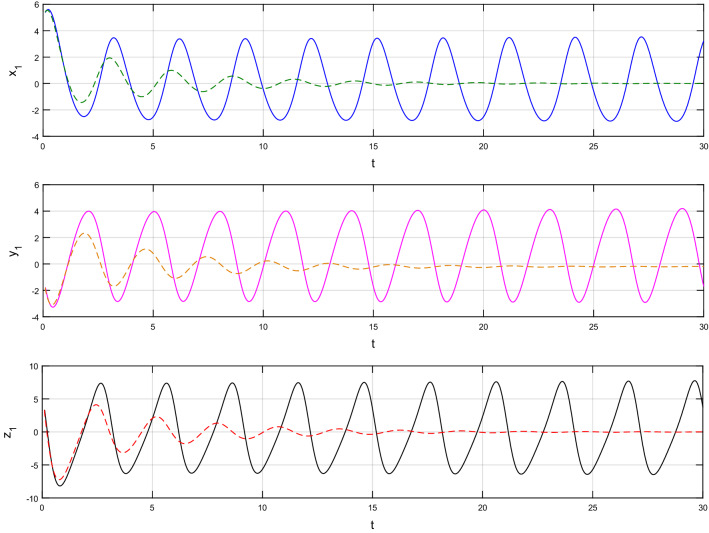
An analysis of the chaotic attractors for this case when *α* = 1 (line) and *α* = 0.90 (dash).

**Figure 6:**
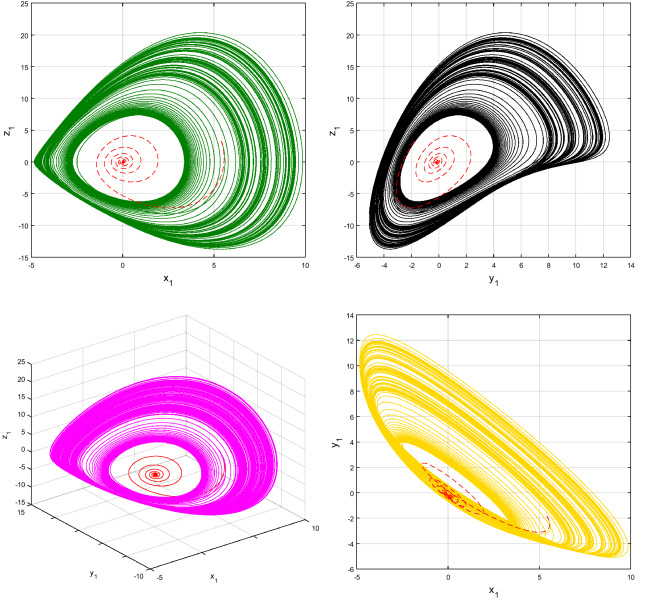
The case's chaotic attractor dynamics, shown in 2D and 3D phase portraits when *α* = 1 (line) and *α* = 0.90 (dash).

**Figure 7:**
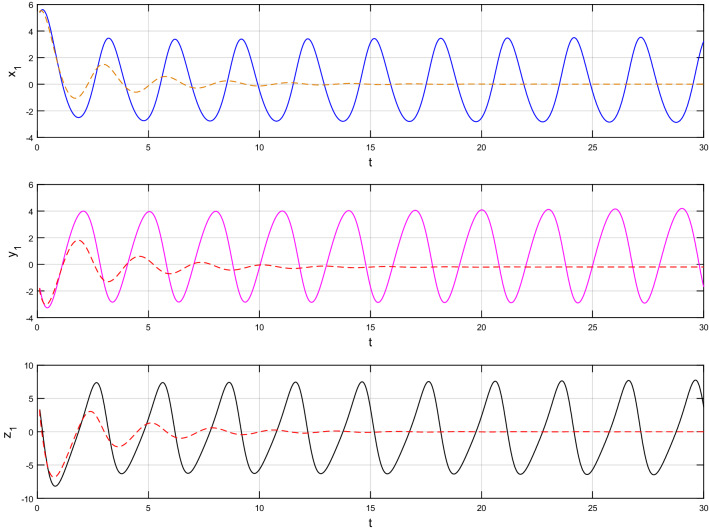
An analysis of the chaotic attractors for this case when *α* = 1 (line) and *α* = 0.85 (dash).

**Figure 8:**
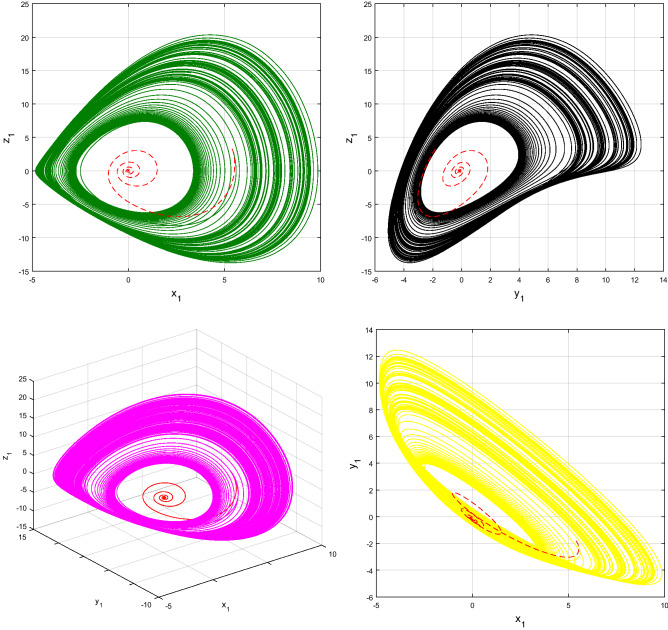
The case's chaotic attractor dynamics, shown in 2D and 3D phase portraits when *α* = 1 (line) and *α* = 0.85 (dash).

**Figure 9:**
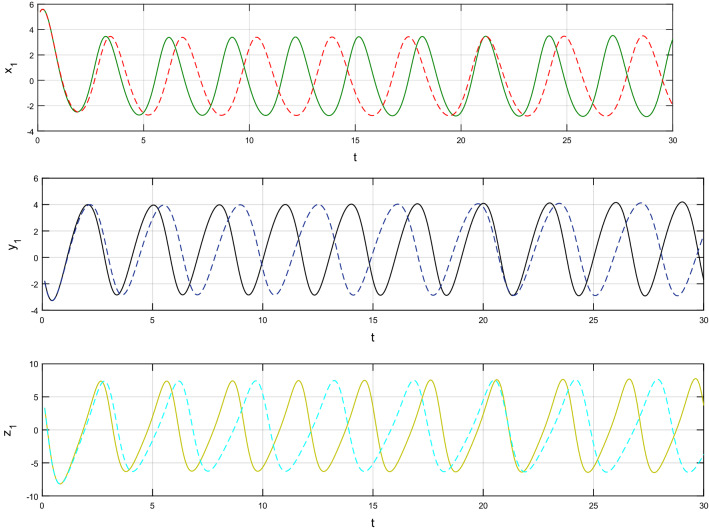
An analysis of the chaotic attractors for this case when *β* = 1 (line) and *β* = 0.95 (dash).

**Figure 10:**
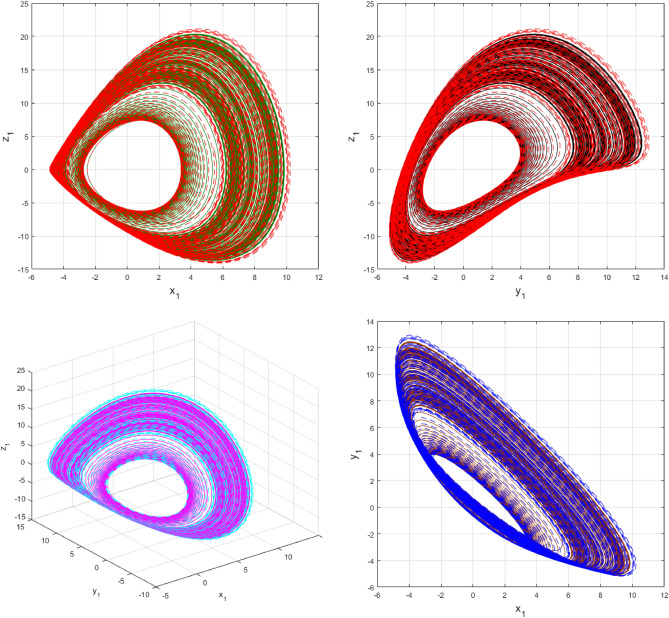
The case's chaotic attractor dynamics, shown in 2D and 3D phase portraits when *β* = 1 (line) and *β* = 0.95 (dash).

**Figure 11:**
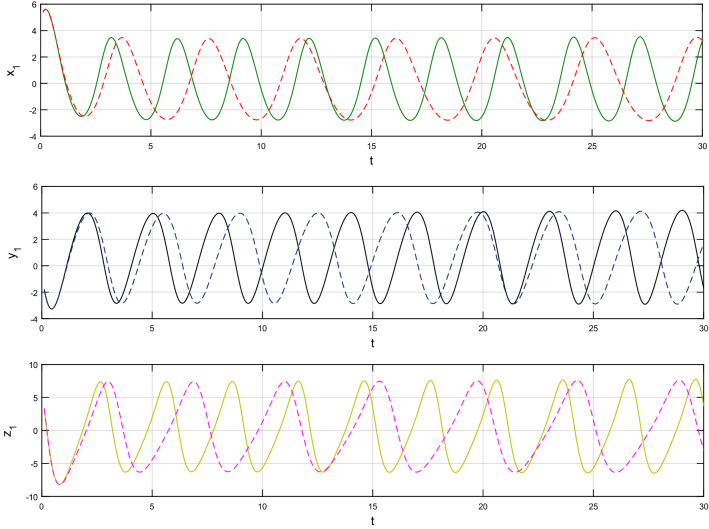
An analysis of the chaotic attractors for this case when *β* = 1 (line) and *β* = 0.90 (dash).

**Figure 12:**
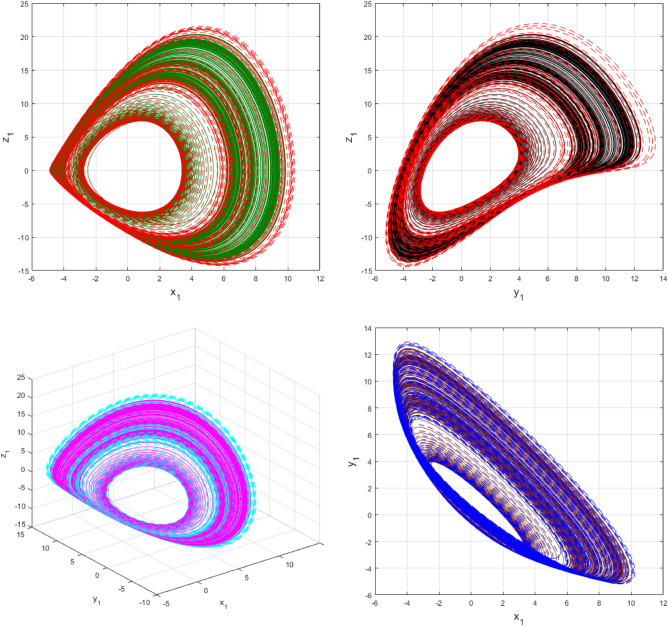
The case's chaotic attractor dynamics, shown in 2D and 3D phase portraits when *β* = 1 (line) and *β* = 0.90 (dash).

**Figure 13:**
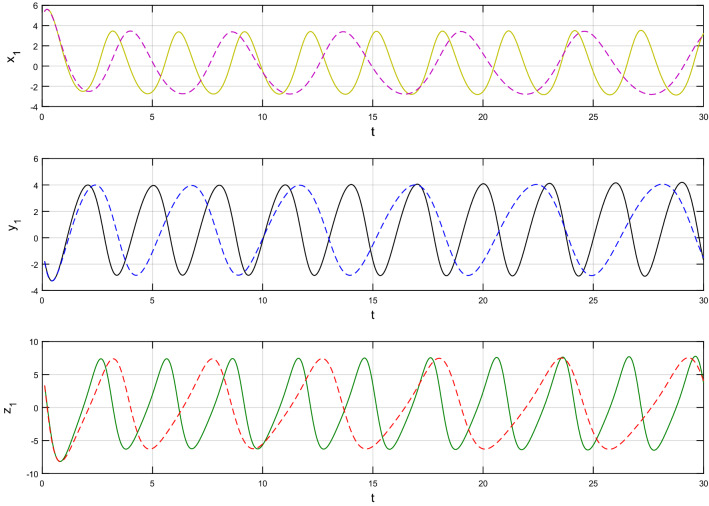
An analysis of the chaotic attractors for this case when *β* = 1 (line) and *β* = 0.85 (dash).

**Figure 14:**
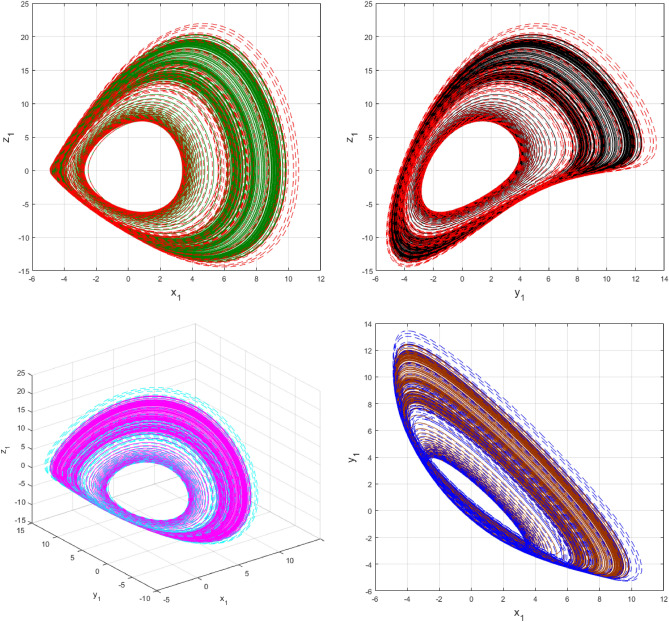
The case's chaotic attractor dynamics, shown in 2D and 3D phase portraits when *β* = 1 (line) and *β* = 0.85 (dash).

Using $\alpha = \beta = 1$ and keeping the other variables constant, Figs. 1 and 2 illustrate the classical behavior of a chaotic attractor. The influence that the fractional parameter $\alpha$ has on the chaotic attractor behavior is seen in Figs. 3, 4, 5, 6, 7, 8. As seen in Figs. 3 and 4, the dynamics converges to its static equilibrium when fractional parameter $\alpha$ is reduced while in the classical/integer order, it always evolve around its equilibrium. Further, it can be noticed that the time period and amplitude of the oscillations reduces by reducing $\alpha$. The impact $\alpha$ on the dynamic of chaotic attractors conforms to the same configuration as that which is shown in Figs. 5, 6, 7, 8. From the figures depicting the effects of effect $\alpha$ on chaotic attractors, perhaps the current fractal-fractional theory provides us with several solutions, rather than one as in classical/integer order. It provides us an alternative, and if we make the necessary adjustments, we may be able to get the best outcome possible by combining the results of our experiments with the theoretical data. Figures 9, 10, 11, 12, 13, 14 illustrates the effect that the fractal dimension $\beta$ has on the dynamics of chaotic attractors. We made the observation that decreasing the value of the fractal dimension parameter $\beta$ causes the chaotic attractor dynamics to persist for a longer period of time while the amplitude approximately remains the same. There are various 2D and 3D phase graphs produced in order to better understand the dynamical behavior of the considered chaotic system. For the purpose of making the differences between them more evident, we compare each figure to the classical order. We can observe the dynamics of the problem-limited cycles and periodic orbits that have been described based on the graphs that have been displayed so far.

## Concluding remarks

This study has been carried out for the chaotic system based on circuit design presented by a three-dimensional chaotic system. The problem is modelled in the form of nonlinear integer order ODEs along with the initial conditions. In addition, theoretical analyses of the problem such as equilibria and dissipation have been calculated while the existence and uniqueness of the solutions have been proved. In order to generalize the classical model, we used a fractal-fractional differential operator of power law. To get the graphical solution, a numerical approach is also stated, after which it is implemented using the MATLAB software for simulations. Several graphs are used to illustrate the strange attractors that the chaotic system produces.

According to our observations, the chaotic system exhibits certain unusual behaviors as it fluctuates. Because of this, the outcomes of the current model can be significantly changed, and since fractional-order parameters and fractal dimensions are involved in the current study to make it more general. In addition, it is obvious from the figures shown above that a reduction in fractional parameter $\alpha$ will result in decrease in amplitude as well as time period of oscillations while a decrease in fractal parameter $\beta$ would result in increase in the periods of the trajectories. Modifying these parameters is most likely to produce results that are comparable to those obtained numerically with the experimental results. It is noteworthy to notice that, when $\alpha = \beta = 1$, we may recover the integer order results from the modified fractal-fractional one.

## Data Availability

The data will be available from the corresponding author upon request.
